# The Origin of Low Contact Resistance in Monolayer Organic Field‐Effect Transistors with van der Waals Electrodes

**DOI:** 10.1002/smsc.202100115

**Published:** 2022-03-07

**Authors:** Ming Chen, Boyu Peng, Radu A. Sporea, Vitaly Podzorov, Paddy Kwok Leung Chan

**Affiliations:** ^1^ Department of Mechanical Engineering The University of Hong Kong Pok Fu Lam Road Hong Kong China; ^2^ Department of Electrical and Electronic Engineering Advanced Technology Institute University of Surrey Guildford GU2 7XH UK; ^3^ Department of Physics and Astronomy Rutgers University Piscataway 08854 NJ USA; ^4^ Advanced Biomedical Instrumentation Centre Hong Kong Science Park Shatin Hong Kong China

**Keywords:** contact resistance, monolayer semiconductor, organic transistors

## Abstract

The successful commercialization of organic field‐effect transistors (OFETs) for advanced integrated organic electronics requires reducing device sizes, which inevitably clashes with the constraints imposed by the contact effects. Herein, it is demonstrated that the contact resistance in OFETs based on monolayer organic semiconductors is extremely low, especially at mild biasing conditions. The contributions of the access resistance and the metal–organic interface resistance are successfully disentangled for the first time. It is shown that, contrary to the conventional view, the contact resistance of monolayer OFETs in the saturation regime exhibits a very weak dependence on the source electrode length. In the monolayer OFETs based on 2,9‐didecyldinaphtho[2,3‐b:2’,3’‐f]thieno[3,2‐b]thiophene (C_10_‐DNTT), a gate‐voltage‐independent access resistivity (2.2 × 10^−2^ Ω cm^2^) at *V*_DS_ = −1 mV is obtained, while the interfacial metal–organic Schottky contact resistance is found to be negligible. The depletion of a diode associated with the metal–organic interface expands with increasing *V*_DS_ and eventually bottlenecks the device performance. Finally, how to overcome such a carrier depletion contact resistance bottleneck and achieve OFETs with outstanding performance are shown. These findings pave the way toward sophisticated organic electronic applications based on the use of monolayer OFETs.

## Graphical abstract

Herein, the contact resistance is studied in monolayer organic transistors with van der Waals electrodes. The contact resistivity reveals no gate‐dependent behavior under low drain‐source bias while the reverse diode in the metal–organic interface becomes dominated when drain‐source bias increases. Herein, a deep insight into the contact resistance of the monolayer organic transistors is provided.

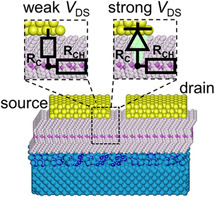

## 1 Introduction

As the core building blocks of organic electronics, organic field‐effect transistors (OFETs) have made impressive progresses in recent decades. The recent realization of OFETs with a high‐charge carrier mobility (*μ*) exceeding 10 cm^2^ V^−1^ s^−1^ has accelerated further development of these devices and provided a solid foundation for fast organic transistors capable of a high‐frequency operation.^[^ ^1^, ^2^, ^3^, ^4^, ^5^ ^]^ Other than fast operating speed, these flexible and highly crystalline organic materials have been shown to be promising in other different applications, including organic logic circuits, memory devices, various sensors, medical applications, and display drivers.^[^ ^1^, ^2^, ^3^, ^4^, ^6^, ^7^, ^8^, ^9^, ^10^, ^11^ ^]^ Solution processing approaches for the growth of high‐quality organic thin films of up to a wafer‐scale size have been reported by several groups.^[^ ^3^, ^12^ ^]^ However, to fully utilize the advantages of highly crystalline organic semiconductors, large‐area deposition is not enough; availability of ultrathin films, or even films with the thickness of down to a single molecular layer, is also very important for reducing the access contact resistance. This is necessary for maintaining the high‐speed operation (especially in the staggered top‐contact/bottom‐gate OFETs), while further shrinking the device size dictated by the overall circuit miniaturization. Recently, we have successfully demonstrated a sub‐thermionic subthreshold swing and a high‐current‐density operation of OFETs based on monolayers of a p‐type organic semiconductor, 2,9‐didecyldinaphtho[2,3‐b:2’,3’‐f]thieno[3,2‐b]thiophene (C_10_‐DNTT), used as the active layer.^[^ ^13^, ^14^ ^]^ In addition, the impactful studies on the monolayer molecular crystals (MMCs) have been conducted broadly. The excellent optical properties of MMCs enable the high‐performance optoelectronics.^[^ ^15^, ^16^ ^]^ Sensitive to the external stimuli owing to the ultrathin feature, the MMCs provide promising candidate for sensor application.^[^ ^9^, ^17^ ^]^ Meanwhile, the ultrathin smooth surfaces of MMCs allow the high‐quality p–n junction and logic circuits.^[^ ^18^, ^19^ ^]^ In this communication, we investigate the origin of the low contact resistance observed in these monolayer OFETs leading to their outstanding performance.

The contact resistance (*R*_C_) in OFETs represents a major roadblock in downsizing these devices. In short‐channel OFETs, a significant fraction of the applied drain‐source voltage (*V*_DS_) may drop on the contact resistance, leading to less efficient, contact‐dominated device operation. Different approaches to a reduction of contact resistance, including the use of thin dielectric layers,^[^ ^2^ ^]^ surface doping,^[^ ^20^ ^]^ or modification of the metal–semiconductor interface, were found to be beneficial.^[^ ^21^ ^]^ Recently, a few groups have suggested a new class of OFETs based on monolayer or bilayer organic semiconductors with van der Waals (vdW) integration of metal electrodes, which are very promising for further reduction of *R*_C_. The rationale behind this approach is based on the fact that the contact resistance consists of two major parts: (i) the metal–semiconductor interface resistance, and (ii) the access resistance.^[^ ^22^ ^]^ In principle, reducing active layer thickness can significantly lower the access resistance.^[^ ^13^, ^23^ ^]^ Most commonly, the contact resistance is extracted by the so‐called transmission line method (TLM), which yields a combined value,^[^ ^2^, ^3^, ^13^, ^20^, ^21^, ^22^, ^23^ ^]^ with the individual contributions of the interface resistance (*R*_I_) and the access resistance (*R*_A_) difficult to disentangle. However, knowing their contributions to *R*_C_ is very valuable, especially in high‐mobility and/or short‐channel OFETs, where the channel resistance (*R*_CH_) could be smaller than *R*_C_. Furthermore, in monolayer OFETs, the low access resistance can easily magnify the depletion effect of *R*_I_ at the metal–organic interface, particularly near the source contact.^[^ ^24^, ^25^ ^]^ Thus, a deeper understanding of the contact effects is highly desired for the performance evaluation of monolayer OFETs in the linear or saturation operation regimes.

In the current work, OFETs’ active layer consists of a 3.9 nm thick monolayer of C_10_‐DNTT grown on silicon dioxide (SiO_2_) by solution shearing. The channel width‐normalized contact resistance (*R*_C_ *W*) is extracted at *V*_DS_ = −1 mV, and a minimum value of 89.9 Ω cm is achieved at the gate voltage *V*_G_ = −80 V. Contrary to the conventional OFETs with thicker active layers, the specific contact resistivity (*ρ*_*C*_) of the monolayer C_10_‐DNTT OFETs shows no gate‐voltage dependence for *V*_DS_ below −1 V, and a low *ρ*_C_ value of 2.2 × 10^−2^ Ω cm^2^ is obtained. This is because the access resistance, *R*_A_, is dominant when the monolayer OFET is operating under a low *V*_DS_ (i.e., *R*_A_ >> *R*_I_). When *V*_DS_ is increased, the contribution of the reverse‐biased Schottky diode at the source–semiconductor interface becomes stronger and eventually starts dominating (i.e., *R*_I_ >> *R*_A_). The sufficiently high reverse bias at the source region would cause carrier depletion and lead to a premature saturation of the source‐drain current (*I*_DS_). When saturation occurs, we confirm that the carriers injected from the source to the channel are restricted by the localized depletion region at the edge of the source, and the dependence of the contact resistance on the source electrode length vanishes. This is a unique property that can only be observed in the transistors with high mobility, ultrathin active layer, and clean metal–organic Schottky contacts. The performance prediction based on different *R*_C_ values can guide further efforts on device miniaturization of these distinct OFETs. Finally, we demonstrate that the reverse bias source depletion can be eliminated by using a thin layer of insulating material with a high dielectric constant to replace the silicon dioxide and operating such a device at lower *V*_DS_.

## 2 Results and Discussion

### 2.1 Fabrication and Electrical Performance of Monolayer OFETs

**Figure 1:**
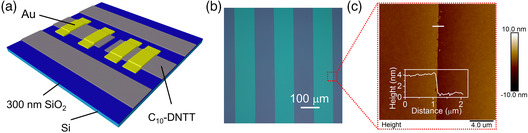
Fabrication of monolayer organic field‐effect transistors (OFETs) with Au contacts by van der Waals integration. a) A schematic illustration of a staggered top‐contact/bottom‐gate OFET structure composed of a patterned monolayer 2,9‐didecyldinaphtho[2,3‐b:2’,3’‐f]thieno[3,2‐b]thiophene (C_10_‐DNTT) (grey) and laminated Au electrodes (yellow). When two or more devices are fabricated on a single stripe, the monolayer semiconductor is scratched between the devices to prevent cross talk. b) A polarized optical microscopy (POM) image of the patterned active layer. The brighter green and the darker grey regions are the C_10_‐DNTT and the bare substrate, respectively. The scale bar is shown in the figure. c) Atomic force microscopy (AFM) topography scan on the edge of the organic semiconductor stripe, as indicated by the red box in panel (b). The inset is a height profile recorded along the white solid line.

Staggered monolayer OFETs incorporate monolayer C_10_‐DNTT with gold (Au) electrodes (**Figure** **1**a). Monolayer organic semiconductor C_10_‐DNTT was deposited by low‐speed blade‐coating technique, as reported earlier.^[^ ^9^, ^13^ ^]^ To eliminate fringe currents and ensure a 1D carrier transport in the channel,^[^ ^26^ ^]^ the C_10_‐DNTT thin films were first patterned: this was achieved via conformal coating with parylene SR and pre‐patterning of this coating by photolithography and dry etching (Figure S1–S3, Supporting Information). To ensure that the transistor's channel is a single crystal, a width of ≈100 μm (also corresponding to channel width) was adopted for each stripe of monolayer C_10_‐DNTT (Figure 1b). It is worth to mention that the parylene ‘tape’ is specifically positioned so that the C_10_‐DNTT stripes and the carrier transport are along the high‐mobility *a*‐axis.^[^ ^13^ ^]^ The synchronous color change of C_10_‐DNTT stripes as the substrate rotates at 45° (Figure S4, Supporting Information) indicates no grain boundaries and separated domains. The thickness and the sharpness of the monolayer stripe were confirmed by atomic force microscopy (AFM) and scanning electron microscopy (SEM) as shown in Figure 1c and S5, Supporting Information. A step of 3.9 nm agrees well with the thickness of C_10_‐DNTT monolayer.^[^ ^9^, ^13^ ^]^ To ensure clean metal–organic contacts, gold was first evaporated on octadecyltrichlorosilane (OTS)‐treated substrate through a Cu‐grid shadow mask (with openings: 38 × 200 μm). The source and drain electrodes were then fabricated by the vdW integration reported earlier by our team and others,^[^ ^13^, ^14^, ^27^ ^]^ in which the rectangular Au patches were transferred onto the C_10_‐DNTT monolayer. It is fascinating that the vdW integration could be applied to large‐area fabrication and compatible with mass production.^[^ ^27^ ^]^ The entire process flow of the electrode integration, from the original substrate to the patterned monolayer C_10_‐DNTT, is shown in Figure S6, Supporting Information. While the width (*W*) of the conduction channel is defined by the stripe width of the patterned semiconductor (Figure 1a), the channel length (*L*) can be chosen during the electrode lamination under the microscope. As discussed later, such a thermal damage‐free formation of metal–organic interface is essential for the observation of the unique properties of the monolayer OFETs.

**Figure 2:**
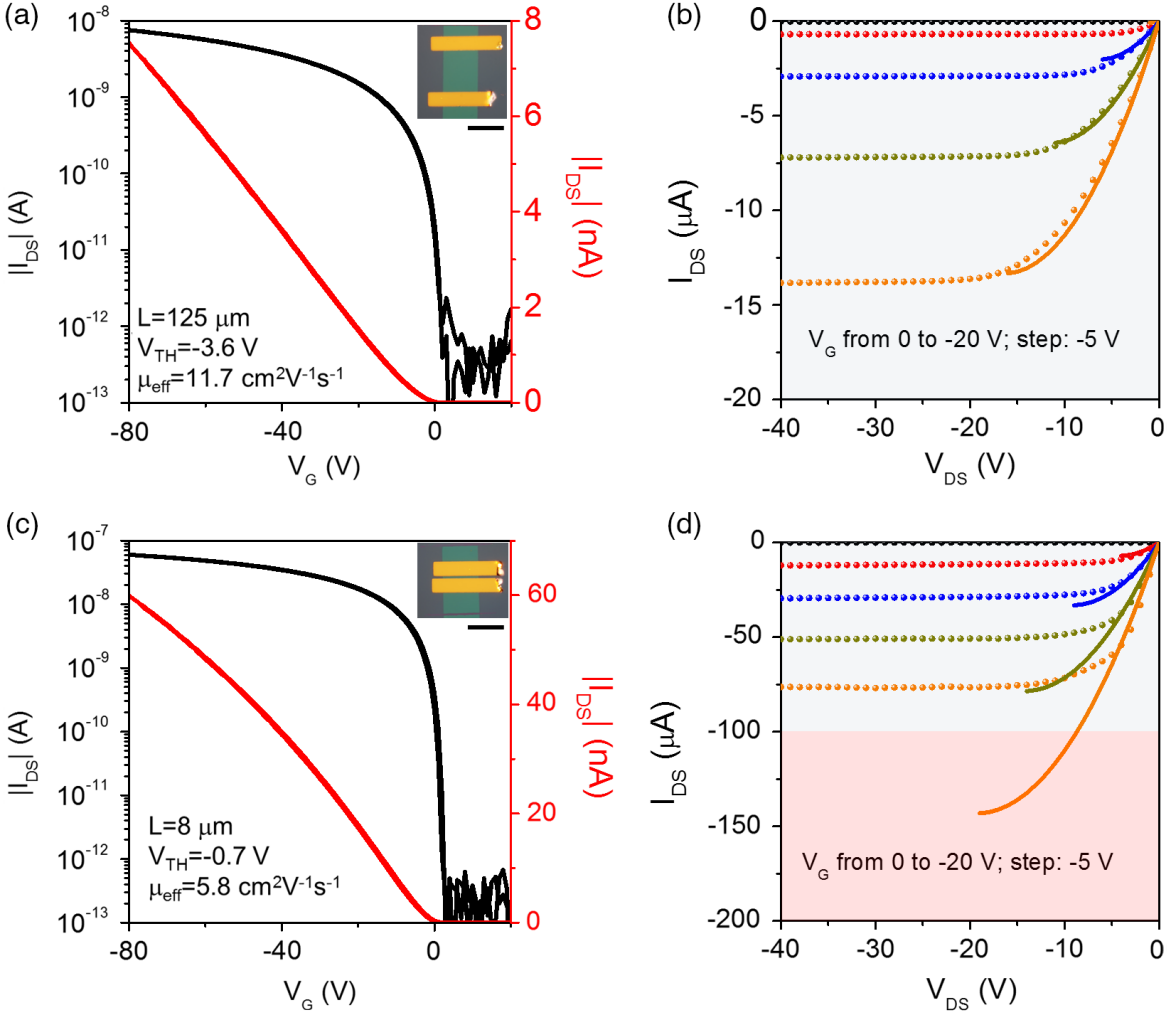
Comparison of the electrical performance of long‐channel and short‐channel monolayer OFETs. a,b) The transfer and output curves of the long‐channel (*L* = 125 μm) OFET. c,d) The transfer and output curves of the short‐channel (*L* = 8 μm) OFET. The transfer curves (a,c) were recorded at *V*_DS_ = −1 mV. The channel lengths, threshold voltages, and mobilities are indicated; the insets show the POM images of the corresponding OFETs. The scale bars in the insets are 100 μm. For the output curves (b,d) of the OFETs, *V*_G_ was varied from 0 to −20 V in steps of −5 V. The experimental results are shown with symbols, while simulations based on gradual channel approximation (GCA) model are shown with solid lines. The grey and pink shadings correspond to the stable and unstable operation regimes, respectively.

**Figure** **2** shows the electrical characteristics of two monolayer OFETs with the channel lengths of 125 and 8 μm. Previously, we have observed that when the width‐normalized current in monolayer OFETs exceeds ≈1 A m^−1^, the devices would start suffering from the Joule heating effects leading to degraded output curves after the first test.^[^ ^13^ ^]^ Thus, we have restricted the applied *V*_G_ and *V*_DS_ during testing to maximum −20 and −40 V, respectively, to avoid detrimental heating effects and allow multiple and repeated testing of the devices without degradation (Figure S7, Supporting Information). Figure 2a–d shows the transfer and output curves of both the long‐channel and short‐channel devices. The polarized optical microscopy (POM) images of the devices are shown as insets in Figure 2a,c. For the *L* = 125 μm device, the transfer curve at *V*_DS_ = −1 mV shows an effective mobility of 11.7 cm^2^ V^−1^ s^−1^ (Figure 2a), which is comparable to the intrinsic value in the later extraction. However, in the *L* = 8 μm device, the effective mobility is 50% lower: 5.8 cm^2^ V^−1^ s^−1^ (Figure 2c). Moreover, a distinct difference appears in the output curves. While the output characteristics of the *L* = 125 μm OFET show a textbook behavior, with *I*_DS_ saturating at $V_{\text{DS}}\geq V_{G}-V_{\text{TH}}$ (*V*_TH_ is the threshold voltage), the *I*_DS_ of the short‐channel device shows an early saturation at *V*_DS_ < *V*_G_ − *V*_TH_. Figure 2b,d shows the experimental results, as well as simulated output curves up to $V_{\text{DS}}=V_{G}-V_{\text{TH}}$ calculated based on the gradual channel approximation (GCA). The difference between them suggests that the potential drop along the channel of the short‐channel monolayer OFETs no longer follows the GCA.

### 2.2 Extraction of Access Resistance in Monolayer OFETs

To understand what causes the premature current saturation in monolayer OFETs, we need to understand the contact effects in these devices. The reduction of channel length from 125 to 8 μm is accompanied by a decrease of the channel resistance *R*_CH_, which accentuates the contact effects. As mentioned briefly in the introduction, the contact resistance of an OFET comprises both the interface resistance, *R*_I_, and the access resistance, *R*_A_, as described by Equation (1)

(1) $$R_{C}=R_{I}+R_{A}$$

The *R*_I_ originates from the metal–organic Schottky barrier, which is highly sensitive to the external applied electrical field. Depending on the direction and magnitude of the field, the current passing through the Schottky contact can be significantly altered, and the current density at the metal–organic junction can be potentially described by Equation (2).^[^ ^22^, ^28^, ^29^ ^]^

(2) $$J=A^{*}T^{2}e^{\frac{-\phi_{B}}{kT}}[e^{\frac{qV}{nkT}}-1]=J_{0}[e^{\frac{qV}{nkT}}-1]$$

where *J* is the current density, A* is the Richard constant, *ϕ*_B_ is the barrier height, *T* is the temperature, *k* is the Boltzmann constant, *q* is the elementary charge, *V* is the application voltage, *n* is the ideality factor, and *J*_0_ is the reversed saturated current density. Specifically, in organic semiconductors with relatively low mobility while comparing with the inorganic one, the current travelling through the contact has shown to be linear proportion to the bulk mobility.^[^ ^30^, ^31^, ^32^, ^33^ ^]^ The *R*_I_ value is given by RI=dVdJAinj, where *A*_inj_ is the charge injection area.

The *R*_A_ arises from the flow of the injected carriers in the “vertical” direction, when they traverse the film's thickness, moving from the metal–semiconductor interface to the conduction channel. The values of *R*_A_ are largely determined by the thickness of the organic semiconductor and the material's conductivity in this direction. However, the *R*_A_ value of well‐packed monolayer OFETs should, in principle, be significantly lower than that of multilayer OFETs or OFETs based on thicker thermally evaporated organic films. The sum of the net contact resistance *R*_C_ and the channel resistance *R*_CH_ would give the total resistance (*R*_T_) of the OFET, as given by Equation (3) and (4).

(3) $$R_{T}=R_{C}+R_{\text{CH}}$$

(4) $$R_{T}W=R_{C}W+R_{\text{CH}}W=R_{I}W+R_{A}W+R_{\text{SH}}L$$

where *R*_SH_ is the sheet resistance of the channel derived from the gradual channel approximation. In single crystal organic semiconductors, the highly ordered molecular structure renders flat transport band, along which the carriers travel with the intrinsic mobility (*μ*_0_). Regarding the single crystal active layer, *R*_SH_ is related to the intrinsic mobility and the gate‐channel capacitance per unit area (*C*_i_) via Equation (5).

(5) $$R_{\text{SH}}=\frac{1}{\mu_{0}C_{i}\left|\left(V_{G}-V_{\text{TH}}\right)\right|}$$

Equation (4) is the main equation used for evaluation of contact resistance by TLM.^[^ ^2^, ^3^, ^13^, ^20^, ^21^, ^22^, ^23^, ^34^ ^]^

**Figure 3:**
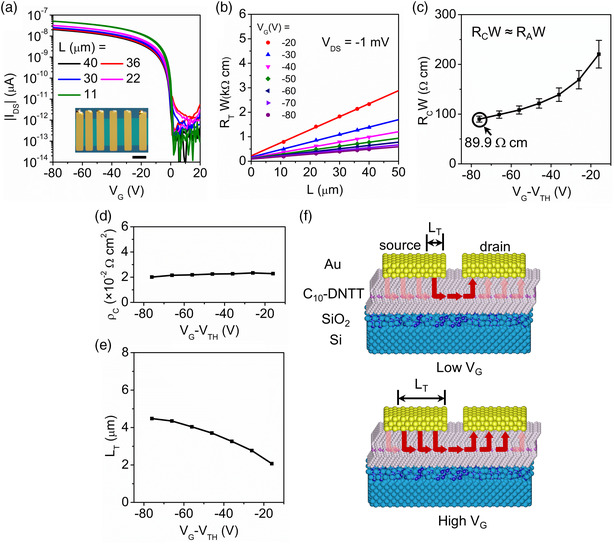
Extraction of the access resistance in monolayer OFETs. a) The transfer curves of a series of transistors of a varied channel length (indicated) prepared on the same single‐crystal stripe of C_10_‐DNTT and measured at *V*_DS_ = −1 mV. The inset shows a POM image of the device series prepared for transmission line measurements: *W* = 100 μm, and *L* = 11, 22, 30, 36, and 40 μm. The scale bar is 50 μm. b) The total resistance as a function of the channel length, recorded at various *V*_G_ (indicated) and fitted with transmission line method (TLM) model. c–e) The contact resistance, the contact resistivity and the carrier injection length versus *V*_G_ − *V*_TH_. f) Schematic diagrams of the current flow in a monolayer OFET at low (upper) and high (lower) *V*_G_. The red arrows represent the dominant current flow; the semitransparent pink arrows represent much weaker injection and flows. The phenyltrichlorosilane molecules are omitted for clarification.

By transferring Au electrodes onto the single crystalline C_10_‐DNTT layer, five OFETs with the channel lengths of 11, 22, 30, 36, and 40 μm are constructed (inset in **Figure** **3**a). The transfer curves of all these OFETs with different channels are measured at a small *V*_DS_ = −1 mV (Figure 3a), and the contact resistance is extracted from the intercept of *R*_T_ *W* (*L*) plot (Figure 3b). Thus, obtained contact resistance *R*_C_ *W* is gate‐voltage dependent, and it reaches the smallest value of 89.9 Ω cm at *V*_G_ = −80 V (Figure 3c). Apart from the contact resistance, we have also determined the sheet resistance (100.3 kΩ sq^−1^ at *V*_G_ = −80 V), the linear‐regime mobility (12.2 ± 0.5 cm^2 ^V^−1 ^s^−1^), and the contact resistivity (*ρ*_C_) (2.2 × 10^−2^ Ω cm^2^) as functions of *V*_G_−*V*_TH_ (Figure 3d and Figure S8, Supporting Information). The definition and the expression for *ρ*_C_ are given in Figure S9, Supporting Information, and the corresponding text in the Supporting Information.^[^ ^35^ ^]^ It is worth noting that both area injection and edge injection could occur in ultrathin semiconductor FETs, as observed in transition metal dichalcogenide devices,^[^ ^36^ ^]^ and TLM is valid for area injection. One major observation here is that *ρ*_C_ is independent of *V*_G_−*V*_TH_ (Figure 3d), which is different from the behavior of conventional OFETs.^[^ ^28^ ^]^ Such a *V*_*G*_‐independent *ρ*_C_ is a unique characteristic of the monolayer OFETs under low *V*_DS_ bias, where *R*_A_ is dominating the *R*_C_.

The carrier injection length, *L*_T_, is an effective length of the metal–organic interface under the contact, adjacent to the contact's edge of width *W*, that defines an effective injection area *L*_T_ *W*, through which the charges are injected from the source to the channel. *L*_T_ can be calculated based on the model derived in the Supporting Information, showing that *L*_T_ should be proportional to $\sqrt{V_{G}-V_{\text{TH}}}$, provided that *ρ*_C_ is constant with *V*_G_ (Equation (S16), Supporting Information). In Figure 3d, no significant dependence on gate bias suggests *ρ*_C_ is constant and dominated by *ρ*_A_, that is, *ρ*_C_ ≈ *ρ*_A_. Thus, calculated *L*_T_ is plotted in Figure 3e. This suggests that when these monolayer OFETs are under low source‐drain bias, the effective charge injection area increases with a stronger gating, as schematically shown in the diagram in Figure 3f. This is attributed to the fact that the contact resistance at low *V*_DS_ is dominated by *R*_A_, while *R*_I_ is negligible.

### 2.3 The Interface Diode Effect in Monolayer OFETs

**Figure 4:**
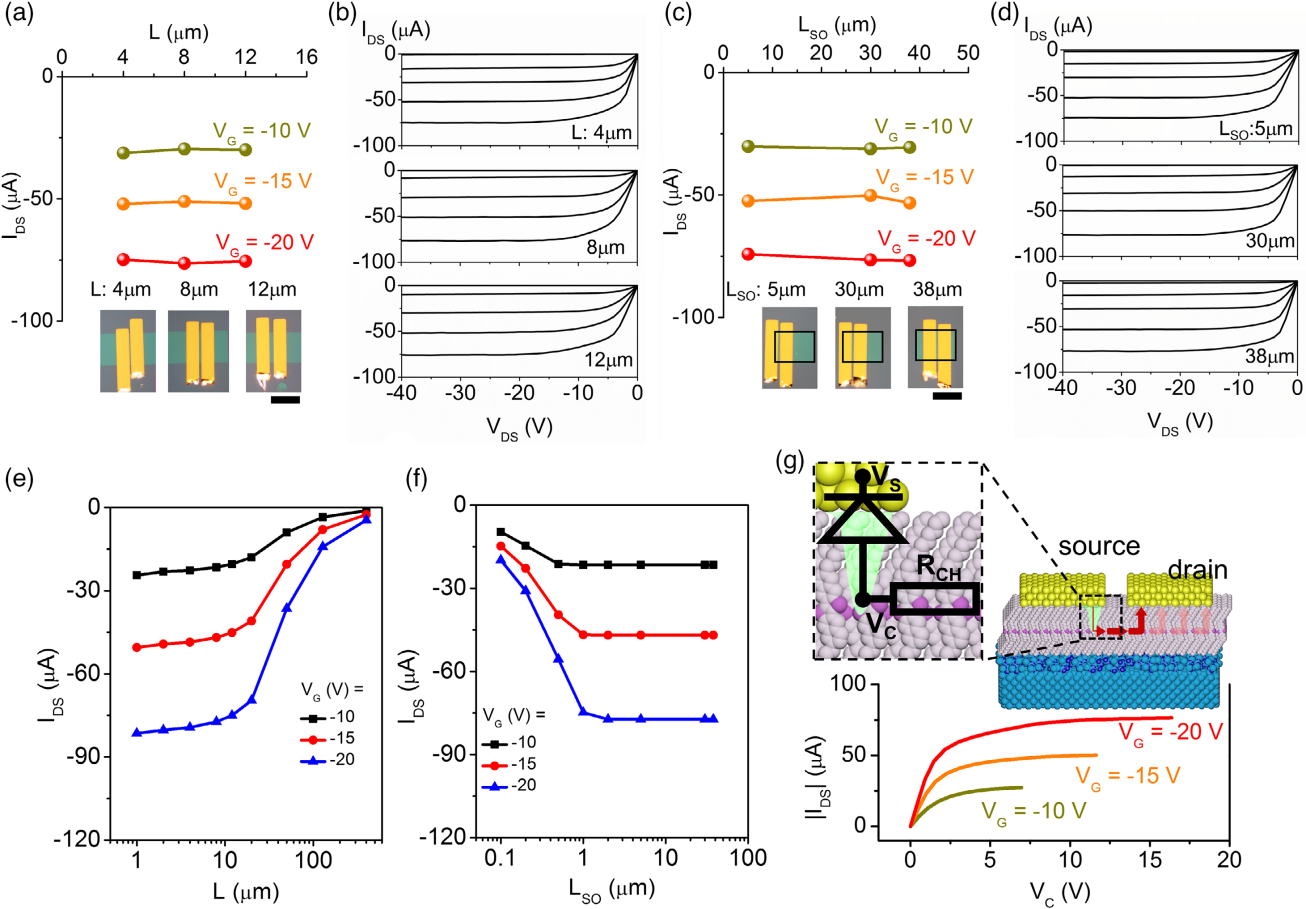
The diode effect in monolayer OFETs. a) The comparison of the source‐drain current values in the transistors with different channel lengths obtained at *V*_DS_ = −40 V. The inset shows the POM images of the monolayer OFETs with different channel lengths (the scale bar is 100 μm). b) The output characteristics of the devices shown in the previous panel. From top to bottom, the output curves correspond to *L* = 4, 8, and 12 μm. c) The comparison of the source‐drain current in the transistors with different source overlap length obtained at *V*_DS_ = −40 V. The inset shows the POM images of the monolayer OFETs with different source overlap lengths (the scale bar is 100 μm). d) The output characteristics of the devices shown in the previous panel. From top to bottom, the output curves correspond to *L*_SO_ = 5, 30, and 38 μm. The added black open boxes indicate the boundary of the active layer in each device. e,f) The results of technology computer‐aided design (TCAD) simulations for the maximum drain current obtained at *V*_DS_ = −40 V and varying channel length *L* and the source overlap length *L*_SO_. g) The drain current versus the contact voltage. The inset is a schematic diagram of the current flow in the monolayer OFET, with the equivalent circuit under source depletion conditions. The green area represents the source depletion region at large *V*_DS_. *V*_S_ and *V*_C_ are, correspondingly, the electric potentials of the source contact and the edge of the current‐carrying conjugated molecular stack at the entrance of the channel (such that *V*_S_−*V*_C_ represents a potential drop across the Schottky barrier at the metal–organic interface).

However, the size of the injection area and low *R*_A_ would become less important when *V*_DS_ increases, which should be particularly obvious in short‐channel devices with significant contact effects. One feature in the short‐channel monolayer OFETs is the premature saturation of *I*_DS_ as shown in the output curves in Figure 2d, where the current saturates at voltages lower than $V_{\text{DS}}=V_{G}-V_{\text{TH}}$. This early saturation is even more obvious at large *V*_G_ (Figure S10, Supporting Information). However, to exclude the thermal damage as mentioned before, here the output curves were recorded under |*V*_G_| not higher than 20 V. The maximum *I*_DS_ currents of the three C_10_‐DNTT monolayer OFETs with the channel lengths of 4, 8, and 12 μm are plotted as a function of *V*_G_ (**Figure** **4**a,b). Although the devices have different channel lengths, they show comparable *I*_DS_ of 75.6 ± 0.8 μA at *V*_G_ = −20 V and *V*_DS_ = −40 V. The output curves of the OFETs in Figure 4b are very similar, except for the linear‐regime region, where the shorter channels result in steeper slopes at low *V*_DS_. Other than the absence of any dependence on the channel length, we have also observed that the maximum *I*_DS_ is independent of the source overlap length (*L*_SO_), which is the length of the interface between the Au source contact and the organic semiconductor (Figure 4c,d). The inset in Figure 4c shows the top‐view optical microphotographs of the devices with *L*_SO_ = 5, 30, and 38 μm, where the C_10_‐DNTT monolayer region is outlined by the black rectangle. The technology computer‐aided design (TCAD) simulation models using Silvaco Atlas are utilized to confirm the observed dependence on *L* and *L*_SO_ (details of the simulations can be found in the corresponding text of Figure S11, Supporting Information). The simulations in Figure 4e show that the contact starts to dominate when the channel length is 10 μm or shorter. Moreover, as shown in Figure 4f, when *V*_DS_ is large (−40 V), the drain current is independent of *L*_SO_, as long as the latter is longer than 1 μm. However, a rapid drop in *I*_DS_ is observed when the gate‐source overlap is less than that. The effective injection length at *V*_G_ = −20 V is in the range of 1 μm. The injection length would reduce to around 0.6 μm when the gate bias is reduced to −10 V. The injection length is further examined in Figure S11, Supporting Information. The plot shows that most of the source electrode does not contribute to the current injection in the monolayer OFETs. In short, the experimental results and simulations suggest that when monolayer OFETs are operating in saturation mode, small‐size electrodes should be utilized for increased density of devices. In addition, one does not need to worry about the device‐to‐device variation due to the differences in the electrode dimensions, because the actual charge injection area is much smaller. Furthermore, as explored in the Section 2.4, the reduction of *L*_T_ suggests that *R*_I_ can no longer be neglected when *V*_DS_ increases.

The lack of dependence of the saturation current on *L*_SO_, as well as the premature saturation of the FETs are related to the depletion of the source contact at high *V*_DS_, occurring in monolayer OFETs. A natural barrier forms at the metal–semiconductor interface due to the energy level mismatch between the highest occupied molecular orbital (HOMO) of C_10_‐DNTT and the work function of Au.^[^ ^7^, ^37^ ^]^ Monolayer OFETs meet the fundamental conditions for forming a source gate transistor (SGT) that comprises a Schottky barrier at the source contact–semiconductor interface, the gate extension beneath the source on the other side of the semiconductor, and an ultrathin semiconductor layer itself.^[^ ^24^, ^25^ ^]^ In an SGT with a large source‐gate overlap, high concentrations of charge carriers would accumulate at high *V*_G_ in the channel region near the semiconductor–dielectric interface. When *V*_DS_ is low, this large amount of charges would provide high conductivity in the channel in the linear regime operation. However, by increasing *V*_DS_ to a certain point, the reversed bias at the source–semiconductor interface would cause a depletion of carriers under the source region (green region in the inset in Figure 4g) and that depleted region would take up most of the applied *V*_DS_. The corresponding energy diagrams are shown in Figure S12, Supporting Information.^[^ ^38^ ^]^ This limits the portion of the voltage dropped on the channel, causing a limitation of *I*_DS_ and an early saturation. As shown in the inset in Figure 4g, when *V*_DS_ increases, the source–semiconductor contact is under a strong reverse bias and will dominate *R*_C_, as compared to the drain–semiconductor contact. Hence, we focus on the source side and evaluate the correlation between the contact voltage (*V*_C_) and *I*_DS_. Since $V_{C}=V_{\text{DS}}-V_{\text{CH}}$, where *V*_CH_ is obtained as *I*_DS_ × *R*_CH_ and *V*_DS_ is known, the *I*_DS_–*V*_C_ relation can be calculated as shown in Figure 4g. It can be clearly seen that the *I*_DS_–*V*_C_ relationship resembles a diode under a reverse bias, where an increasing *V*_C_ (same for *V*_DS_) would not lead to an increase in *I*_DS_.

### 2.4 Contact Resistance and Performance Prediction for Monolayer OFETs

**Figure 5:**
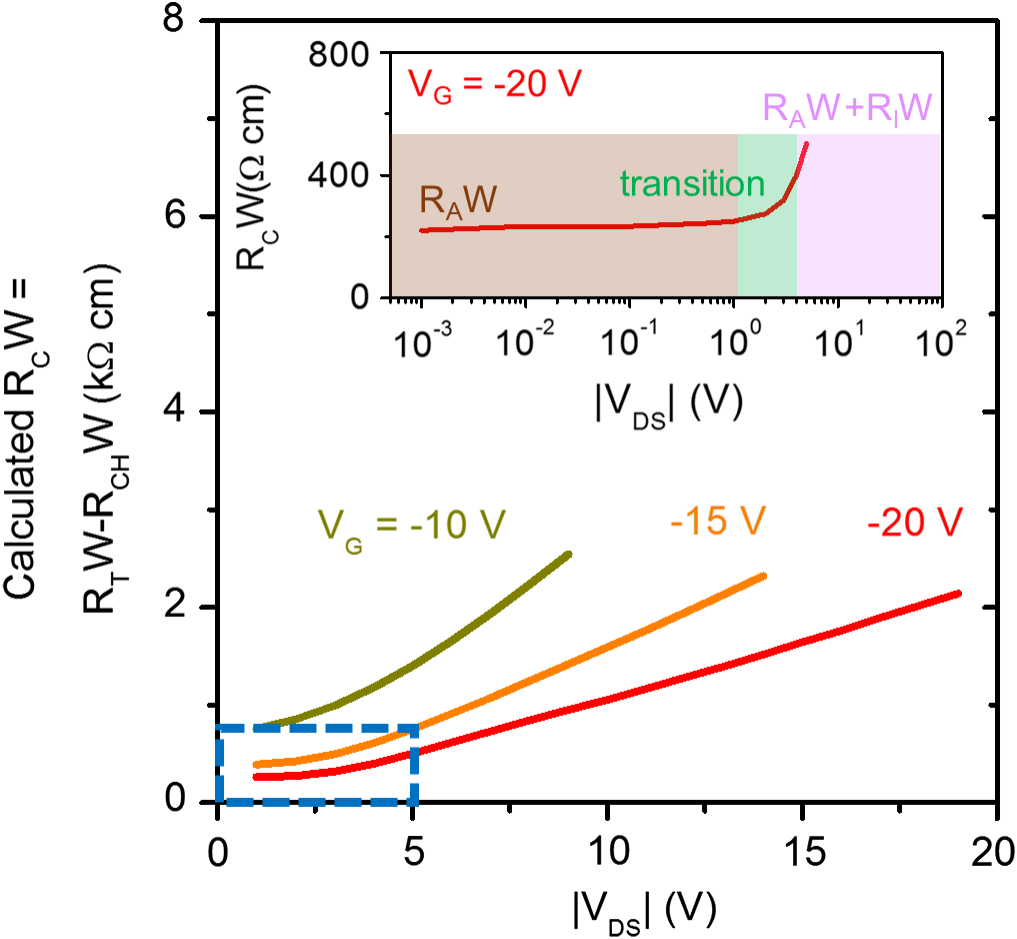
Calculation of the contact resistance in monolayer OFETs at high *V*_DS_. The calculated *R*_C_ *W* versus *V*_DS_. The inset shows the *R*_C_ *W* at small *V*_DS_ and *V*_G_ = −20 V, corresponding to the part of the curve outlined by the blue dashed box. The contact resistance at *V*_DS_ no larger than −1 V was extracted via TLM (Figure S13–S15, Supporting Information).

Based on the *I*_DS_–*V*_C_ correlation observed in Figure 4g, the values of *R*_C_ under different *V*_DS_ and *V*_*G*_ were calculated and plotted in **Figure** **5**. One can see that there is a transition region in *V*_DS_, separating the regime of *R*_C_ governed only by *R*_A_ and the regime where *R*_C_ is governed by *R*_A_ + *R*_I_ (the calculations based on TLM extraction could be found in Figure S13–S15, Supporting Information). The TLM is utilized when *V*_DS_ is no greater than −1 V, as shown in the brown region in the inset. At low *V*_DS_, the *V*_DS_‐independent *R*_C_ implies that the *R*_A_ is dominating and the constant *ρ*_A_ can be attributed to the alkyl chain of the C_10_‐DNTT. We note that achieving a low *R*_A_ (and a low *R*_C_) at low *V*_DS_ requires the mobility of the material to be reasonably high, while at the same time the metal–semiconductor interface to be clean and defects free, as observed in other 2D material systems.^[^ ^27^, ^39^ ^]^ When *V*_DS_ is increased, the contact resistance also increases and becomes dominant by the interface resistance between the source and the semiconductor (i.e., *R*_C_ ≈ *R*_I_). Based on the variation of the contact resistance as a function of *V*_DS_ and *V*_G_, we can predict the performance of monolayer OFETs with different channel lengths (see Figure S16, Supporting Information). The difference in slopes at low *V*_DS_ (also in Figure 4b) results from the total resistance variation for disparate channel lengths. Notably, the contact resistance study in the bottom‐contact configuration is appealing and has achieved impressive progresses with thermally evaporated organic semiconductors.^[^ ^1^, ^2^, ^40^ ^]^ The compatibility of solution shearing is limited by the crystal growth on the bottom‐contact structure.

### 2.5 Mitigation of Depletion Effect in Monolayer OFETs

**Figure 6:**
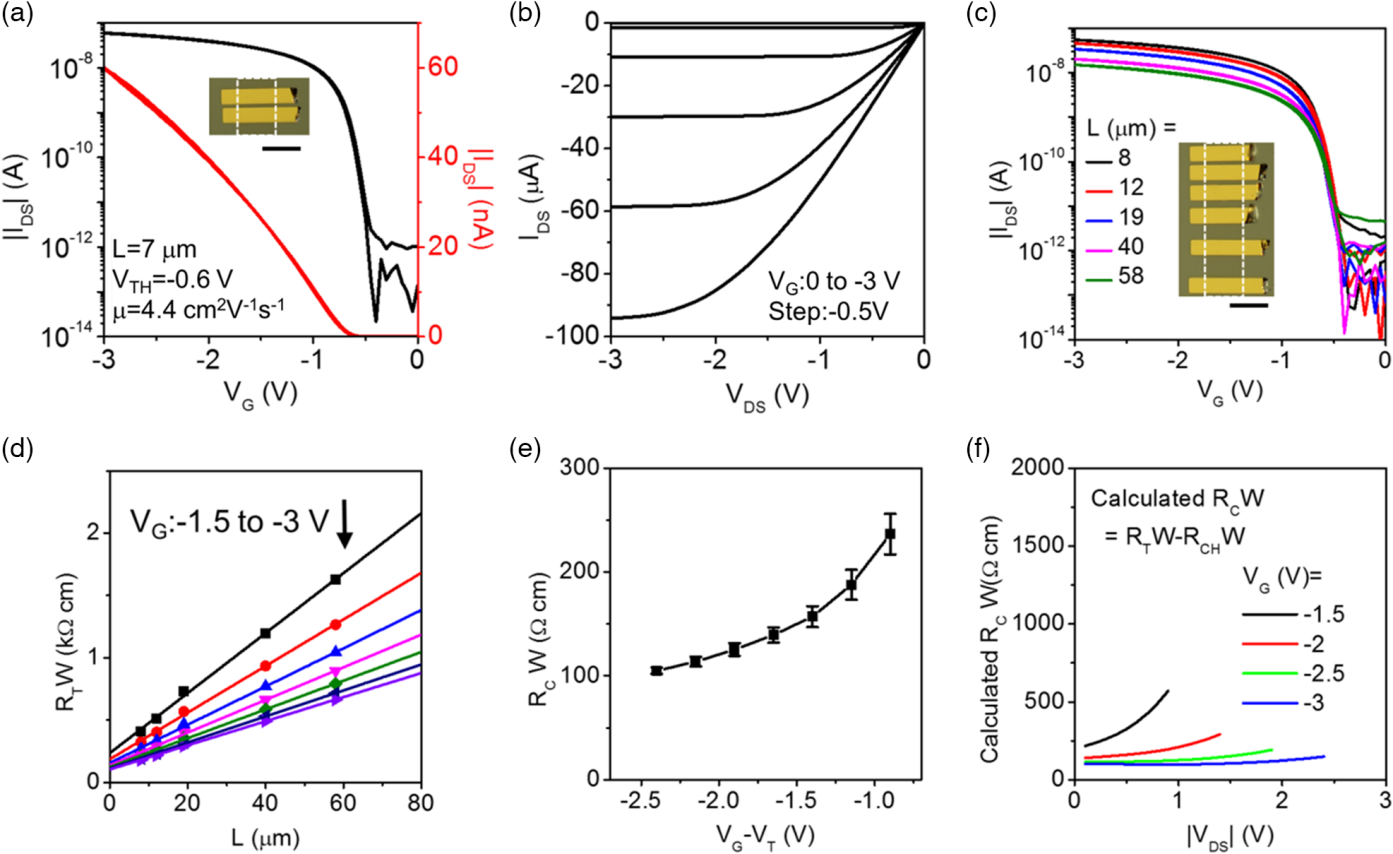
Addressing the problem of premature current with high‐*κ* dielectrics. a) The transfer and b) the output characteristics of a monolayer C_10_‐DNTT OFET using HfO_2_ gate dielectric. The transfer curve was recorded at *V*_DS_ = −1 mV, while the output curves were recorded by sweeping *V*_DS_ from 0 to −3 V. The inset in (a) shows a POM image of the measured short‐channel device (the scale bar is 100 μm). The white dash box shows the outline of the semiconducting layer. c) The transfer curves of the monolayer OFETs with different *L* (recorded at *V*_DS_ = −1 mV). The POM image of the devices is shown in the inset (the scale bar is 100 μm). The organic semiconductor is outlined with a white dash box. d) The TLM fitting. The black arrow shows that *V*_G_ is increasing from −1.5 to −3 V from the top to the bottom curve. e) The extracted *R*_C_ *W* versus *V*_G_−*V*_TH_ obtained from the intercept of the curves in panel (d) with the ordinate axis. f) The calculated *R*_C_W versus *V*_DS_ at different *V*_G_. The contact resistance is determined as *V*_C_/*I*_DS_, where the *V*_C_ is obtained as *V*_DS_−*V*_CH_.

To minimize the depletion effect occurring due to a reverse bias developing across the source–semiconductor interface and to preserve the normal saturation in monolayer OFETs, one can take the most straightforward approach of reducing *V*_DS_ by using a high‐*κ* gate dielectric. To implement this, we have replaced SiO_2_ with 20 nm thick hafnium oxide (HfO_2_) grown by atomic layer deposition (ALD). The capacitance per unit area of our HfO_2_ films was measured to be 450 nF cm^−2^ (Figure S17, Supporting Information). The C_10_‐DNTT monolayer OFETs fabricated directly on HfO_2_ show an effective mobility of 4.4 cm^2^ V^−1^ s^−1^ (**Figure** **6**a, for *L* = 7 μm), and an intrinsic mobility of 10.1 cm^2^ V^−1^ s^−1^ calculated from TLM fitting (Figure 6c,d). The slightly reduced intrinsic mobility in the OFETs using the high‐*κ* gate dielectric is consistent with the general trend observed in thin‐film FETs.^[^ ^41^, ^42^, ^43^ ^]^ This could be attributed to Coulomb scattering, phonon scattering, surface roughness, and Fröhlich polarons. *I*_DS_ saturates at *V*_DS_ values equal to *V*_G_−*V*_TH_, that is, without a premature saturation (Figure 6b). The contact resistance extracted by the TLM at *V*_DS_ = −1 mV and plotted as a function of *V*_G_−*V*_TH_ is shown in Figure 6e. The lowest value of 105 Ω cm is achieved at *V*_G_ = −3 V. In contrast to the monolayer OFETs on SiO_2_, the calculated *R*_C_ *W* shows only a mild variation as *V*_DS_ increases (Figure 6f). The suppression of early saturation is due to the reduction of *V*_DS_ bias and lower saturation current in the device. This finding illustrates that if a high‐*κ* dielectric has surface roughness and surface energy compatible with the solution shearing methodology used for the fabrication of these monolayer OFETs, it can be employed to address the problem of premature saturation in these devices without sacrificing the low contact resistance.

## 3 Conclusion

In summary, the contact resistance *R*_C_ was extracted in monolayer OFETs. At low *V*_DS_ of no greater than −1 V, *R*_C_ is dominated by the access resistance *R*_A_. The dominant role of the access resistance in this regime is verified by the gate‐voltage‐independent access resistivity (2.2 × 10^−2^ Ω cm^2^). With increasing the source‐drain voltage, the monolayer OFETs undergo a source depletion originating from the energy level mismatch between the work function of Au contact and the HOMO edge of C_10_‐DNTT. Under high *V*_DS_, depletion of the source occurs, and *R*_C_ becomes dominated by *R*_I_, with a negligible *R*_A_. The *I*_DS_ is limited by the reverse saturation current of the Schottky contact. The origin and the voltage dependence of *R*_C_ give a window for monolayer OFET electrical performance evaluation. Finally, by decreasing the driving *V*_DS_ via the use of high‐*κ* gate dielectrics, we demonstrate mitigation of the source depletion effect, while preserving the low contact resistance in the monolayer OFETs. These results not only offer substantially deeper insights into the origin of the contact resistance in monolayer OFETs but can also provide valuable guidance to device downscaling efforts in the future.

## 4 Experimental Section

4.1

4.1.1

##### Growth and Patterning of Monolayer C_10_‐DNTT

Two substrates were used in the current work. The SiO_2_/Si substrates were from Namkang Hi‐Tech Co., Ltd. For the high‐*κ* substrates, the HfO_2_ was grown on the Si wafer by ALD. The monolayer C_10_‐DNTT was deposited by the blade‐coating method as reported previously.^[^ ^13^, ^44^ ^]^ A parylene ‘tape’ was used to pattern the monolayer C_10_‐DNTT. The substrates were hydrophobically treated for the following parylene ‘tape’ peeling off. Parylene SR of 500 nm was deposited (Specialty Coating Systems Inc.) followed by the conventional photolithography and etching (Diener Electronic GmbH + Co. KG) process to create the parylene patterns (Figure S1, Supporting Information). The parylene ‘tape’ was peeled off and laminated on the monolayer C_10_‐DNTT surface. The patterns of C_10_‐DNTT formed after removal of the parylene “tape.”

##### Characterization and Measurement of Monolayer C_10_‐DNTT

The POM images were taken from polarized microscope (Nikon Eclipse LV100N). The AFM images were obtained by Bruker MultiMode 8 system. The Au lamination method was reported previously.^[^ ^13^, ^33^ ^]^ The electrical tests were carried out by semiconductor parameter analyzer B1500 (Keysight). The mobility was extracted with the following equation.
(6)

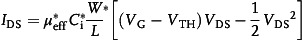




Here, *C*_i_ of 10.6 nF cm^−2^ for 300 nm silicon dioxide is used. The mobility is effective mobility including the contact effect. The similar method has been employed elsewhere.^[^ ^45^ ^]^

## Conflict of Interest

The authors declare no conflict of interest.

## Supporting information

Supplementary Material

## Data Availability

The data that support the findings of this study are available from the corresponding author upon reasonable request.
