# Supplementary material for: The Origin of Low Contact Resistance in Monolayer Organic Field‐Effect Transistors with van der Waals Electrodes
Source: Small Sci. 2022 Mar 7;2(6):2100115. doi: 10.1002/smsc.202100115 (PMC11935811; doi:10.1002/smsc.202100115)
Supplement: Supplementary file 1 — Supplementary Material [file SMSC-2-2100115-s001.pdf]

WILEY-VCH

Copyright WILEY-VCH Verlag GmbH & Co. KGaA, 69469 Weinheim, Germany, 2021.

Supporting Information

The Origin of Low Contact Resistance in Monolayer Organic Field-Effect Transistors with van der Waals Electrodes

*Ming Chen, Boyu Peng, Radu A. Sporea, Vitaly Podzorov and Paddy Kwok Leung Chan**

section S1. Fabrication and characterization

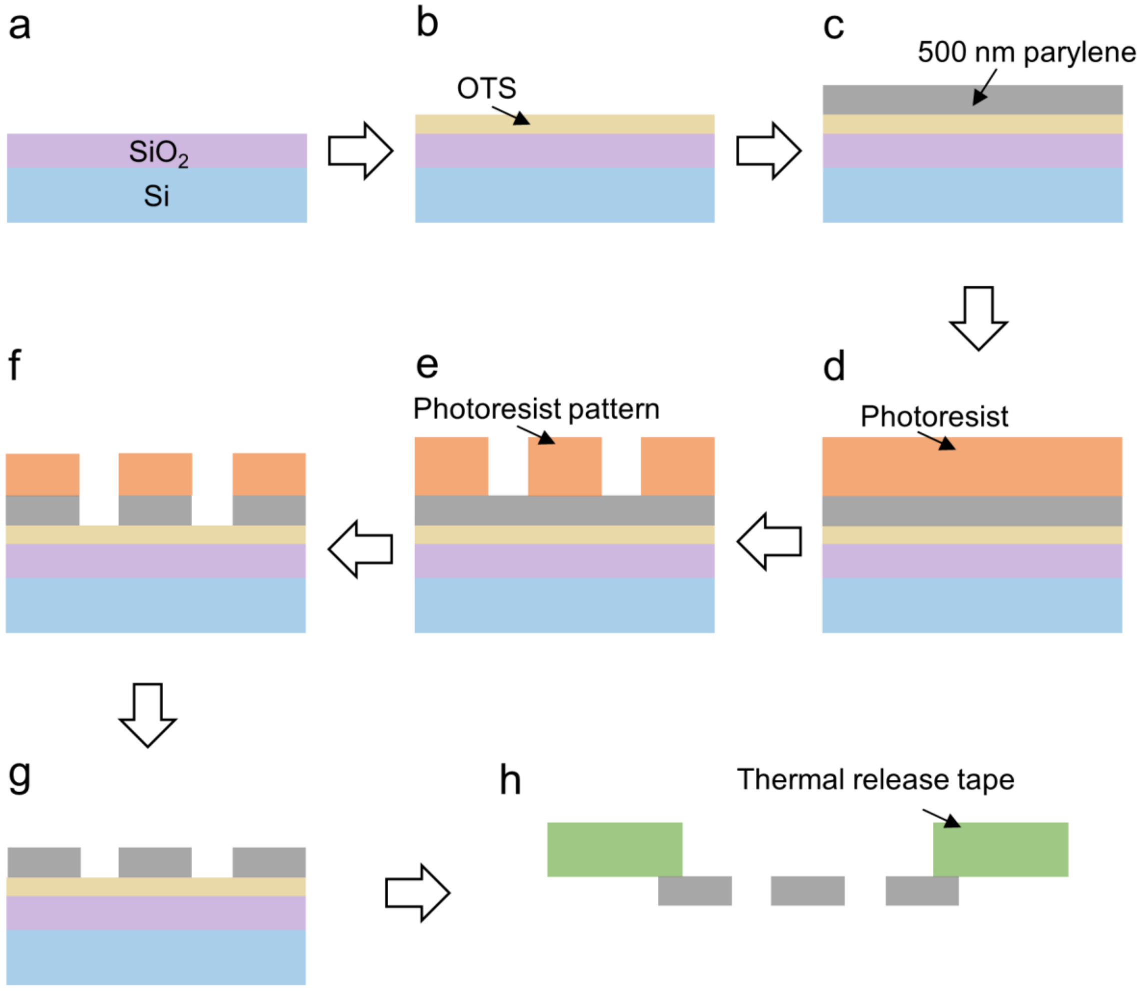

Figure S1. Schematic diagram of the fabrication flow of the parylene 'tape'. (a) The silicon substrate with 300 nm thermally grown SiO_2 is used for the preparation of the parylene 'tape'. (b) The substrate is hydrophobically treated with a self-assembly-monolayer of OTS. (c) 500 nm parylene SR is deposited. (d) A photoresist of AZ nLof 2020 is spun coated on the surface of parylene with the main spin rate of 3000 rpm. (e) The photoresist is patterned by UV-light exposure and development process. (f) The unwanted parylene is removed by reactive ion etching process. The photoresist has much larger thickness of ~2 μm than that of the parylene (500 nm). Therefore, the photoresist can protect the desired parylene patterns well. (g) Removal of the photoresist. (h) The freestanding parylene 'tape' is created by lifting the patterned parylene with the thermal release tape.

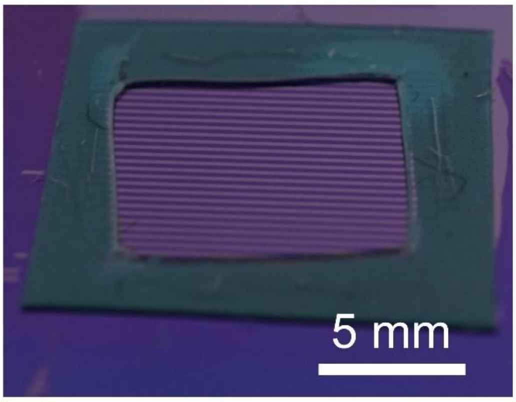

Figure S2. Photograph of parylene ‘tape’ for the organic semiconductor patterning. The parylene ‘tape’ is fabricated by traditional photolithography and etching process on a 500 nm parylene film (Figure S1). The scale bar is shown in the figure. Inside the middle square, the white parallel stripes are the parylene. They serve like tapes to stick and remove the organic semiconductors. The monolayer would cling on the parylene stripes. The purple stripes are the openings and used to later form stripes of organic semiconductors. The patterned parylene ‘tape’ is laminated on the continuous C_{10}-DNTT layer. After removal of the parylene ‘tape’, the continuous C_{10}-DNTT is patterned into narrow strips.

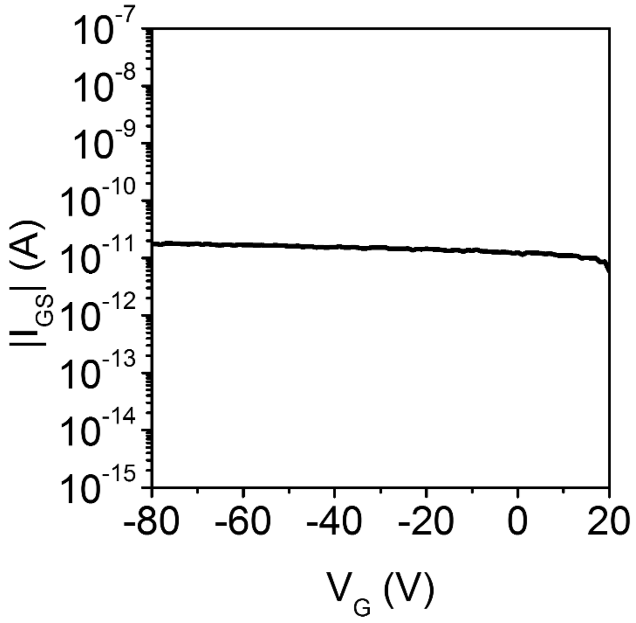

Figure S3. Leakage current test on the patterned organic semiconductor. The current at the whole V_G operation range is ~10 pA, indicating the suppression of the leakage current.

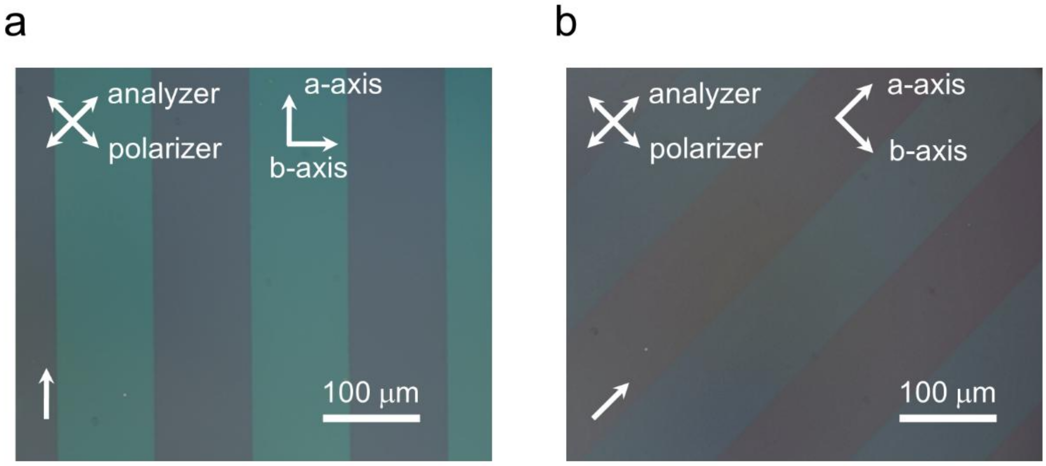

Figure S4. (a) The POM image of patterned C_{10}-DNTT. (b) The POM image of (a) with a substrate rotation angle of 45°. The direction of polarizers and crystal axes are indicated in top of the figures. The white arrows in bottom left denote the shearing direction while the scale bars are shown in the bottom right.

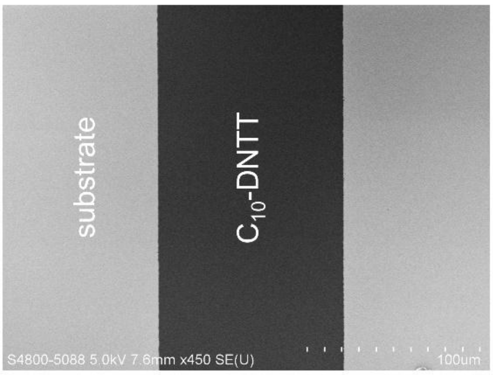

Figure S5. SEM image of the patterned monolayer semiconductors. The bright and dark regions are corresponding to the substrate and C_{10}-DNTT, respectively. The scale bar is shown in the figure.

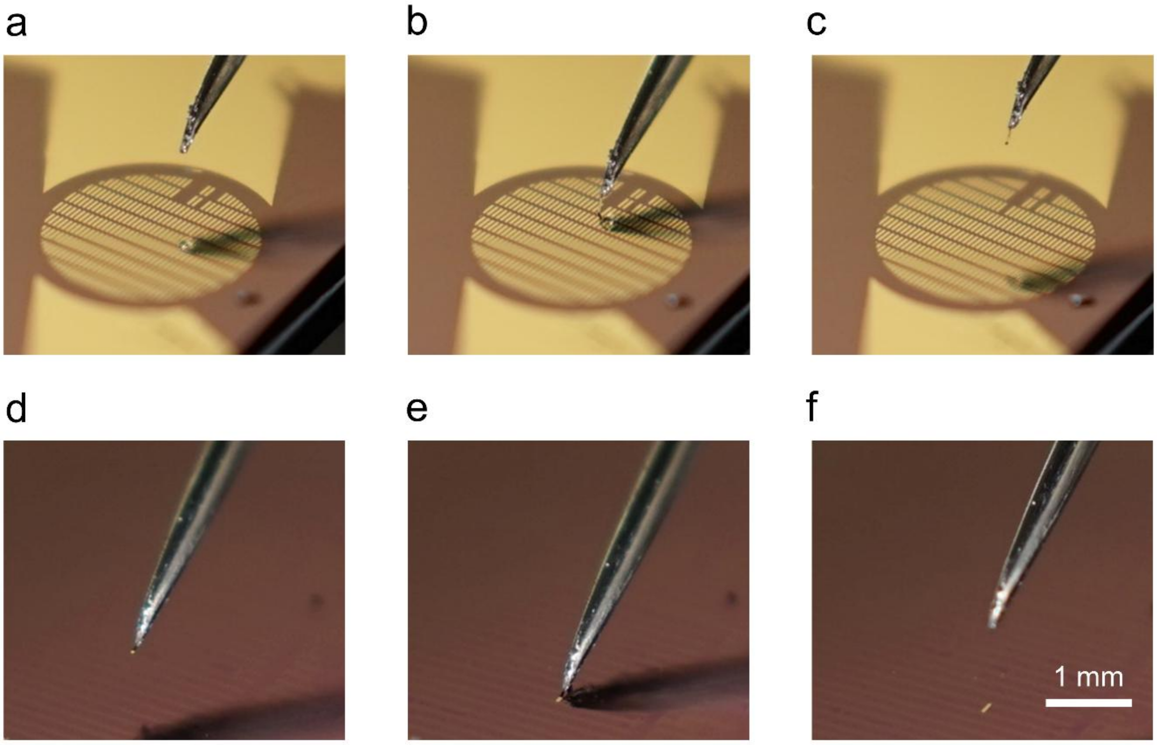

Figure S6. Van der Waals integration flow. (a) The needle is approaching the electrodes under the microscope. The needle is coated with Gallium Indium (GaIn) as the function of glue to stick the Au electrodes. (b) One electrode is lifted by the tip of the needle. (c) The electrode is removed from the substrate and adhere to the needle. (d) The needle with electrode is moving toward the patterned monolayer C_{10}-DNTT. (e) The electrode is placed on the patterned semiconductor. (f) Remove the needle. The van der Waals integration forms between the Au electrodes and the semiconductors. Similar transfer methods have been employed by other groups.[1,2]

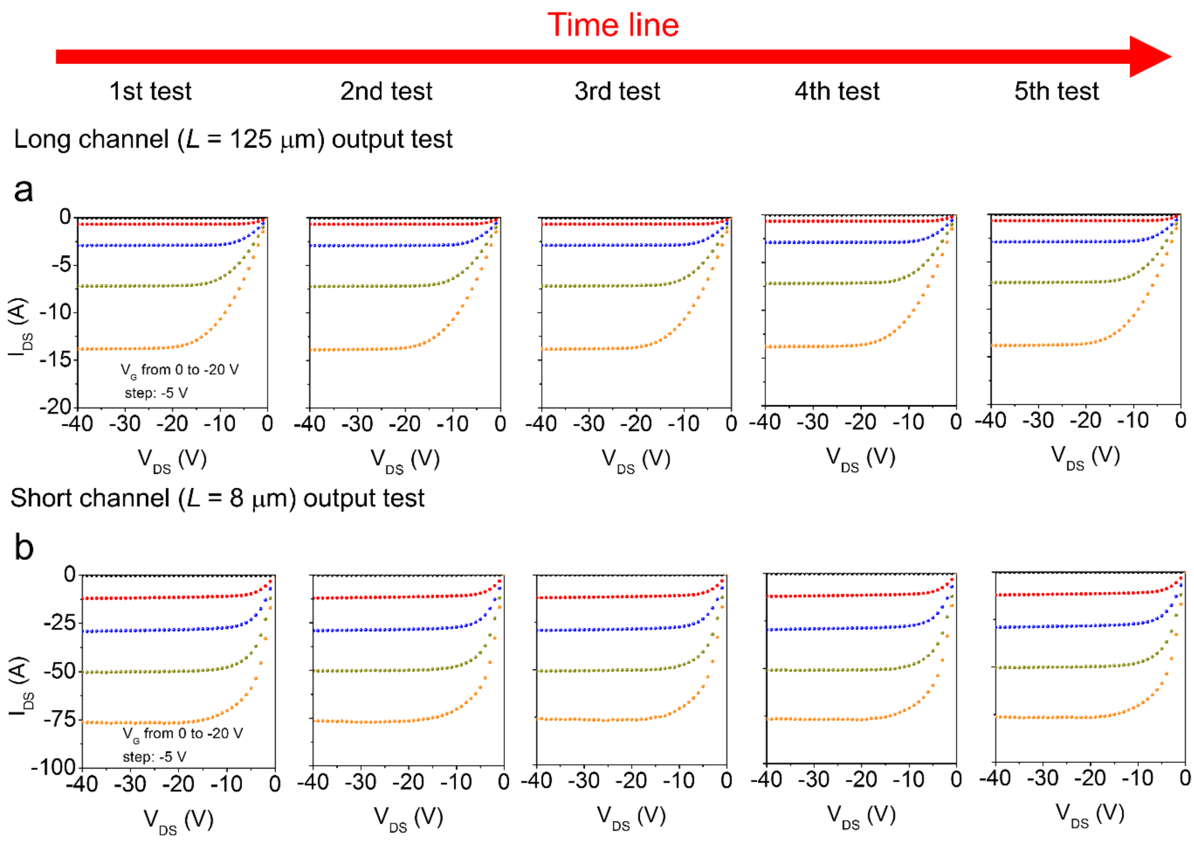

Figure S7. The repeated output tests. (a) The repeated output tests of long channel device (L = 125 μm). (b) The repeated output tests of short channel device (L = 8 μm). The red arrow indicates the time. Both devices were tested for five times.

section S2. Contact resistance study

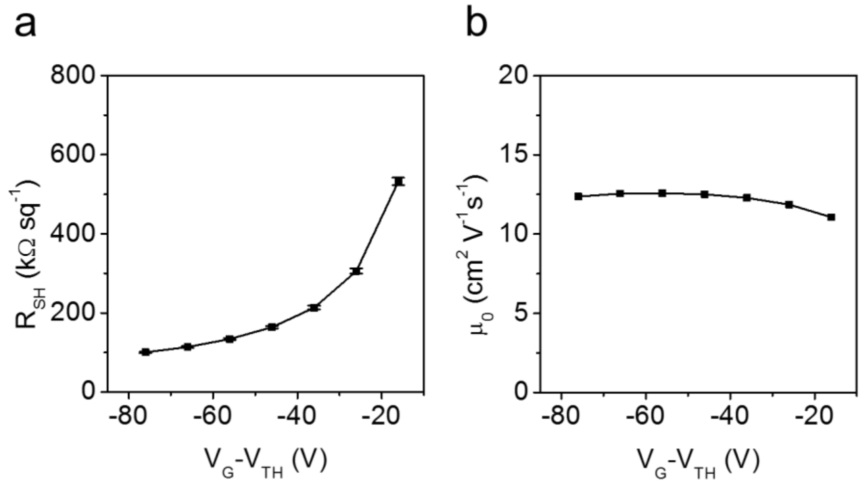

Figure S8. The extraction of parameters from the TLM. The parameters of (a) R_{SH} and (b) μ_0 are plotted with respect to V_G - V_{TH}.

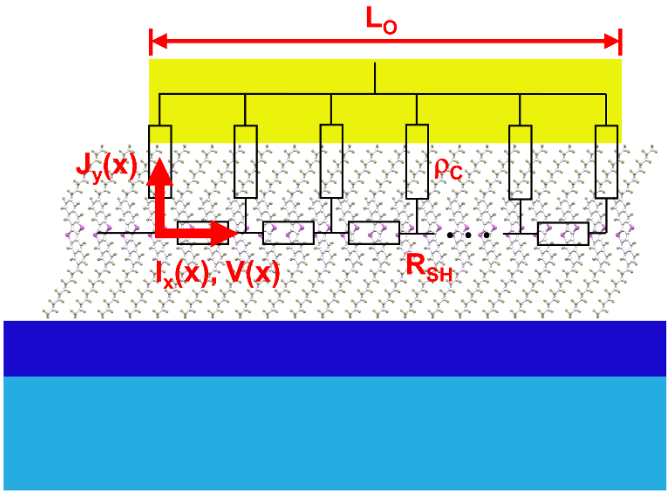

Figure S9. The differential elements of the overall contact resistance. The resistance in the horizontal direction is separated into infinite small differential resistances.

Contact resistance derivation and analysis

The in-depth analysis on contact resistance takes the overall resistance as differential elements,[3] as shown in the mixed joint circuits in Figure S9. The current flow across the contact includes both the vertical and horizontal transports. Jung *et al.* provided the detailed derivation based on the mixed joint circuit. The mixed circuit overlapped with the device structure is drawn in Figure S9. Here J_y (x), I_x (x), V (x) are the current density in y direction, the current in x direction, the voltage in x direction. The J_y (x), I_x (x) and V (x) have positive values for convenience. Assuming the electrode has the potential of 0 V, Kirchhoff's law gives the following mathematical equations, i.e.

(i). when the current passes through the horizontal resistance (the current flow through the resistance is horizontal), the voltage difference on the resistance is equal to the product of the moving current of $I_x(x)$ and the resistance of $R_{SH}dx/W$:

$$V(x) - V(x + dx) = I_x(x)R_{SH}\frac{dx}{W} \quad \text{(S1)}$$

(ii). when the current passes through the vertical resistance (the current flow through the resistance is vertical), the voltage drop on the resistance is equal to the product of the moving current of $J_y(x)Wdx$ and the resistance of ρ_C/Wdx :

$$V(x) = J_y(x)Wdx\frac{\rho_C}{Wdx} \quad \text{(S2)}$$

(iii). The current at position of x is the sum of the current flows integrated from position x to the overlap length:

$$I_{\mathrm{x}}(x) = W\int_{x}^{L_{\mathrm{O}}} J_{\mathrm{y}}(x)\,dx \quad \text{(S3)}$$

Where R_{SH}, ρ_{C} and L_{O} are the sheet resistance, contact resistivity in y direction and the overlap length between the contact and semiconductor for a common gate. The Equations S1 to S3 yield a differential formula of J_{y} (x):

$$\frac{d^2 J_{\mathrm{y}}(x)}{dx^2} = \frac{R_{\mathrm{SH}}}{\rho_{\mathrm{C}}} J_{\mathrm{y}}(x) \quad \text{(S4)}$$

The general solution of Equation S4 is:

$$J_{\mathrm{y}}(x) = J_{\mathrm{y0}} cosh\,(\frac{x}{L_{\mathrm{T}}}) + J_{\mathrm{y1}} sinh\,(\frac{x}{L_{\mathrm{T}}}) \quad \text{(S5)}$$

where:

$$L_{\mathrm{T}} = \sqrt{\frac{\rho_{\mathrm{C}}}{R_{\mathrm{SH}}}} \quad \text{(S6)}$$

The $V(x)$ and $I_{\mathrm{x}}(x)$ could be derived from the general solution and the J_{y1} is determined from Equation S1 when $x = 0$:

$$J_{y1} = -J_{y0} tanh\left(\frac{L_O}{L_T}\right) \quad \text{(S7)}$$

Consequently, $J_{\mathrm{y}}(x)$ is expressed as:

$$J_{\mathrm{y}}(x) = J_{\mathrm{y0}}\left[cosh\,(\frac{-x}{L_{\mathrm{T}}}) + tanh\,(\frac{L_{\mathrm{O}}}{L_{\mathrm{T}}})\,sinh\,(\frac{-x}{L_{\mathrm{T}}})\right] \quad \text{(S8)}$$

The boundary condition is set by the current flow continuity in the conduction channel and the semiconductor-electrode overlap region:

$$I_{\mathrm{DS}} = \frac{W}{L}\frac{1}{R_{\mathrm{SH}}}(V_{\mathrm{DS}} - 2\rho_{\mathrm{C}} J_{\mathrm{y0}}) = I_{\mathrm{x}}(x=0) = WL_0 J_{\mathrm{y0}} tanh(\frac{L_{\mathrm{O}}}{L_{\mathrm{T}}}) \quad \text{(S9)}$$

One can obtain the expression of J_{y0} as:

$$J_{y0} = \frac{V_{\mathrm{DS}}}{\left[R_{\mathrm{SH}} L_{\mathrm{T}} L tanh(\frac{L_{\mathrm{O}}}{L_{\mathrm{T}}}) + 2\rho_{\mathrm{C}}\right]} \quad \text{(S10)}$$

Therefore:

$$I_{\mathrm{x}}(x) = WL_{\mathrm{T}}J_{\mathrm{y0}}\left[sinh\left(\frac{-x}{L_{\mathrm{T}}}\right) + tanh\left(\frac{L_{\mathrm{O}}}{L_{\mathrm{T}}}\right) cosh\left(\frac{-x}{L_{\mathrm{T}}}\right)\right] \quad \text{(S11)}$$

$$V(x) = \rho_{\mathrm{C}}J_{\mathrm{y0}}\left[cosh\left(\frac{-x}{L_{\mathrm{T}}}\right) + tanh\left(\frac{L_{\mathrm{O}}}{L_{\mathrm{T}}}\right) sinh\left(\frac{-x}{L_{\mathrm{T}}}\right)\right] \quad \text{(S12)}$$

The contact resistance is given as:[3]

$$R_{\mathrm{C}} = \frac{V(x=0)}{I_{\mathrm{x}}(x=0)} = \frac{\rho_{\mathrm{C}}}{WL_{\mathrm{T}}tanh\left(\frac{L_{\mathrm{O}}}{L_{\mathrm{T}}}\right)} \quad \text{(S13)}$$

Note that from Equation S13, one can easily tell that the overall resistance is equivalent to a single resistance with the dimensions of W and L_{T}*tanh* $(L_{\mathrm{O}}/L_{\mathrm{T}})$. Considering the L_{O} (~ 38 μm) is significantly larger than the L_{T}, which reduces the *tanh* $(L_{\mathrm{O}}/L_{\mathrm{T}})$ term to 1, the dimensions turn to W and L_{T}, namely,

$$R_{\mathrm{C}} = \frac{\rho_{\mathrm{C}}}{WL_{\mathrm{T}}} \quad \text{(S14)}$$

Given that,

$$R_{SH} = \frac{1}{\mu_0 C_i |(V_G - V_{TH})|} \quad \text{(S15)}$$

One can obtain:

$$L_T = \sqrt{\frac{\rho_C}{R_{SH}}} = \sqrt{\rho_C \mu_0 C_i |(V_G - V_{TH})|} \quad \text{(S16)}$$

$$R_C W = \frac{\rho_C}{L_T} = \frac{\rho_C}{\sqrt{\rho_C\, \mu_0 C_i (V_G - V_{TH})}} = \sqrt{\frac{\rho_C}{\mu_0 C_i |(V_G - V_{TH})|}} \quad \text{(S17)}$$

At low V_{DS} (V_{DS} much small than V_{G}-V_{TH}), given the dominance of ρ_{A} and the sheet resistance expression, we have:

$$L_T = \sqrt{\rho_A \mu_0 C_i |(V_G - V_{TH})|} \quad \text{(S18)}$$

$$R_C W \approx R_A W = \sqrt{\frac{\rho_A}{\mu_0 C_i |(V_G - V_{TH})|}} \quad \text{(S19)}$$

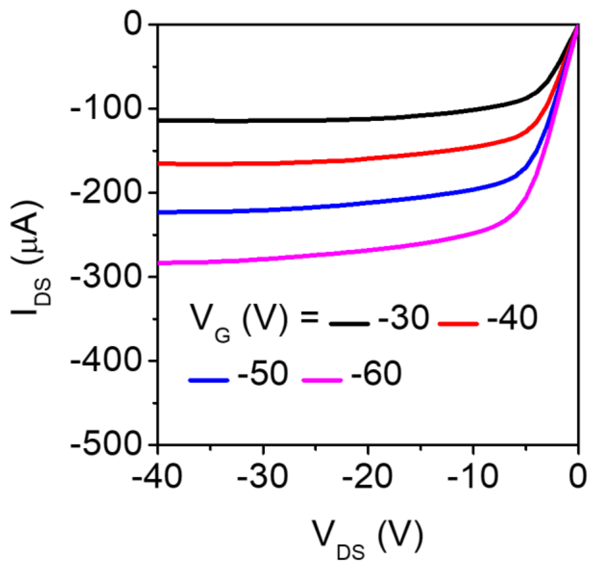

Figure S10. Early saturation at high V_G. The V_G is from -30 V to -60 V with a step of -10 V while the V_{DS} is swept from 0 V to -40 V.

TCAD simulations

Device simulations were performed with Silvaco Atlas v.5.28.1.R using the following parameters to match the measured data: relative permittivity of the insulator: 3.9; relative permittivity of the semiconductor: 3.4; hole mobility parameter: 7.5 cm^2 V^{-1} s^{-1}; semiconductor band gap: 2.55 eV; semiconductor affinity: 2.93 eV; conduction band density of states at 300 K: 10^{20} cm^{-3}; valence band density of states at 300 K: 10^{20} cm^{-3}; source and drain contact work function 5.13 eV to obtain effective equilibrium band offset of 0.35 eV at the contact; field-dependent barrier lowering parameter: 2.7 nm. A range of channel lengths, source-gate overlaps and semiconductor thicknesses have been simulated. In practice, the semiconductor is likely to have anisotropic transport properties which have not been modelled in this study. As such, the simulations fit the measurements for a modelled semiconductor thickness of 10 nm.

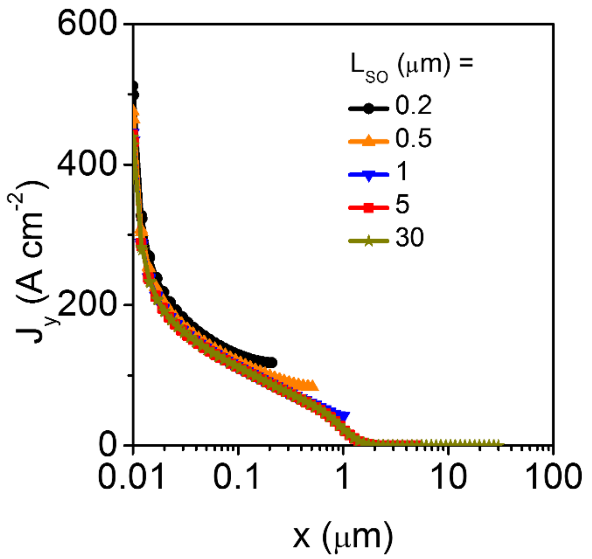

Figure S11. Hole current density at the source contact-semiconductor interface. The current density is plotted at each position along the source, with x = 0 μm representing the edge of the source nearest the drain. The highest current density is seen close to this edge of the source. Resistive drop in the accumulation layer under the source electrode reduces the potential drop across the semiconductor to zero at x > 1.5μm, thus preventing injection from that area of the source.

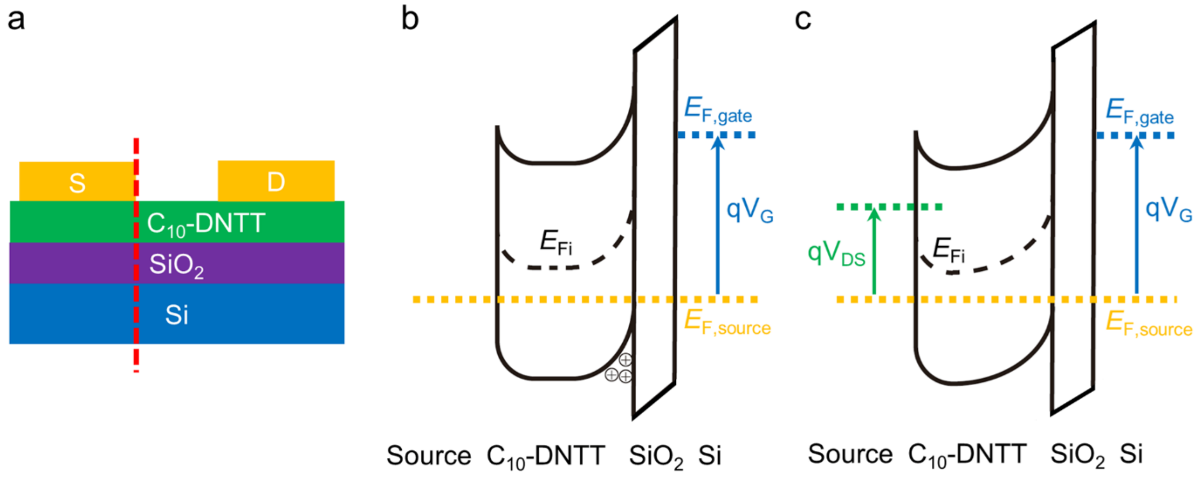

Figure S12. (a) The schematic diagram of the staggered monolayer organic transistor. (b) The energy diagram along the red dash line in (a) without the application of V_{DS}. (c) The lift of V_{DS} increases E_F, which surpasses the intrinsic E_F. The semiconductor has low hole carrier concentration, indicating the carrier depletion.

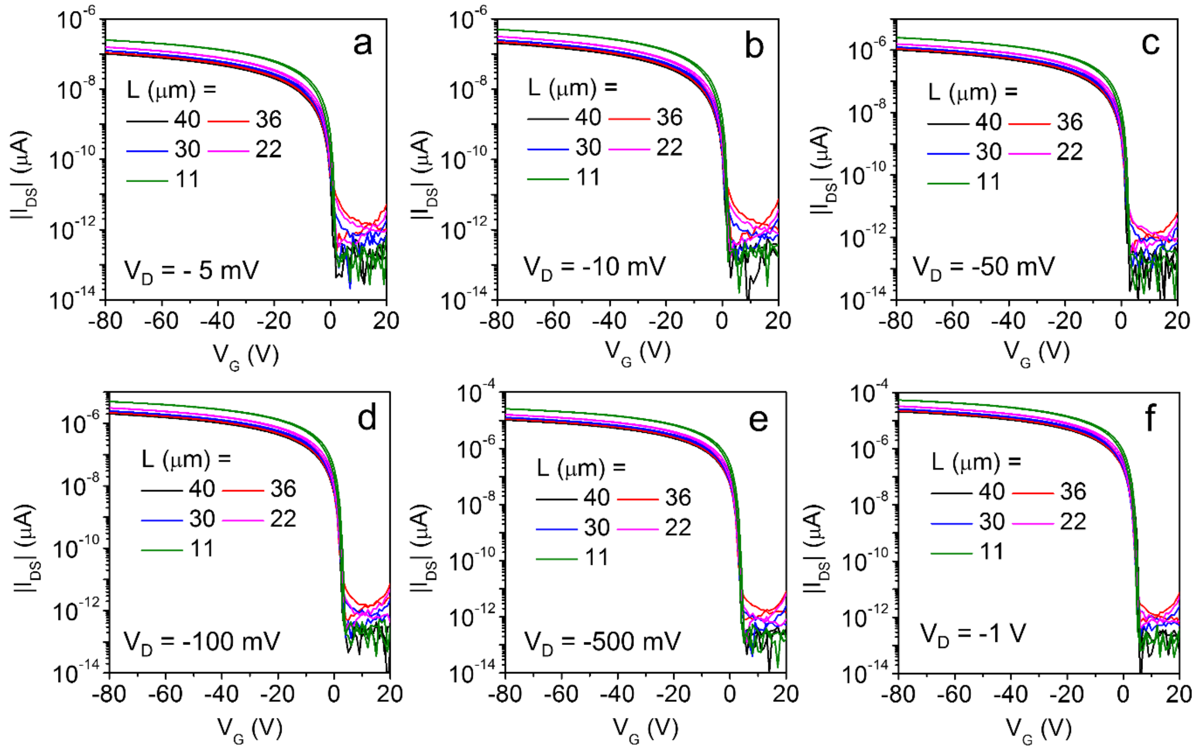

Figure S13. The transfer curves measured at different V_{DS} and different L. The transfer curves are measured at a V_{DS} of (a) -5 mV, (b) -10 mV, (c) -50 mV, (d) -100 mV, (e) -500 mV and (f) -1 V, respectively.

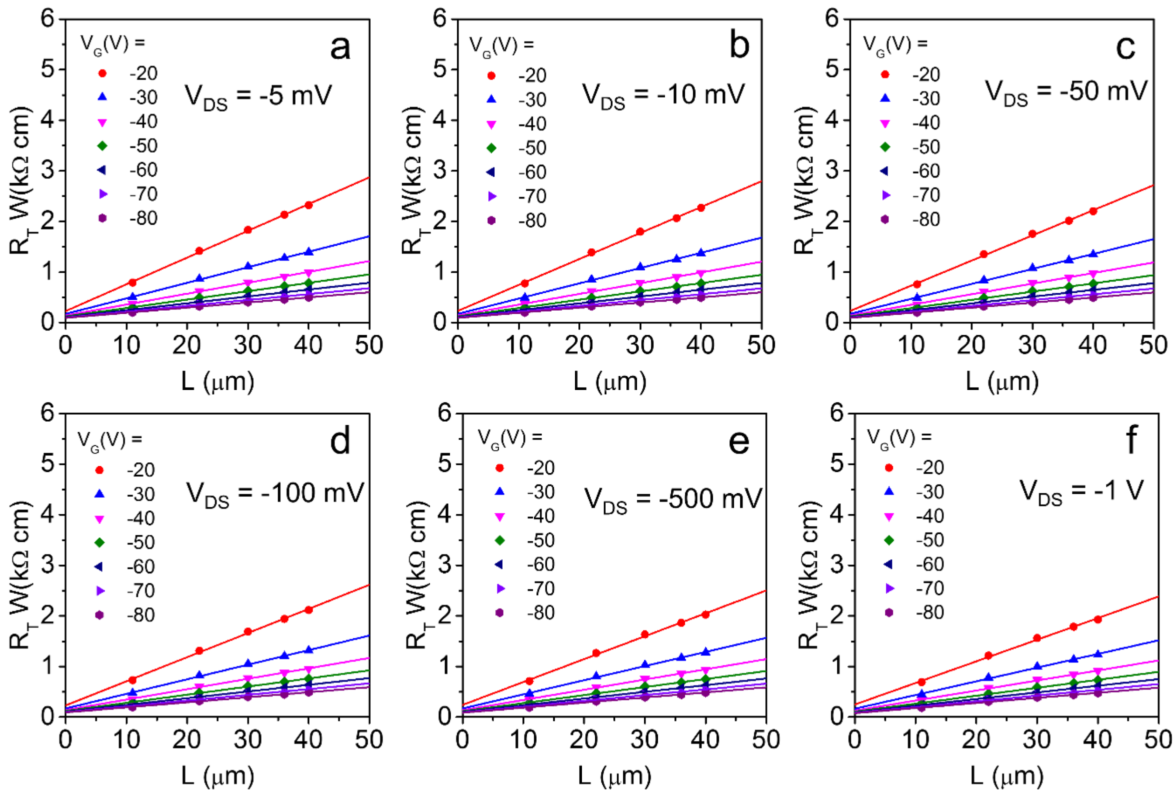

Figure S14. The transmission line method fitting. The V_{DS} are (a) -5 mV, (b) -10 mV, (c) -50 mV, (d) -100 mV, (e) -500 mV and (f) -1 V, respectively.

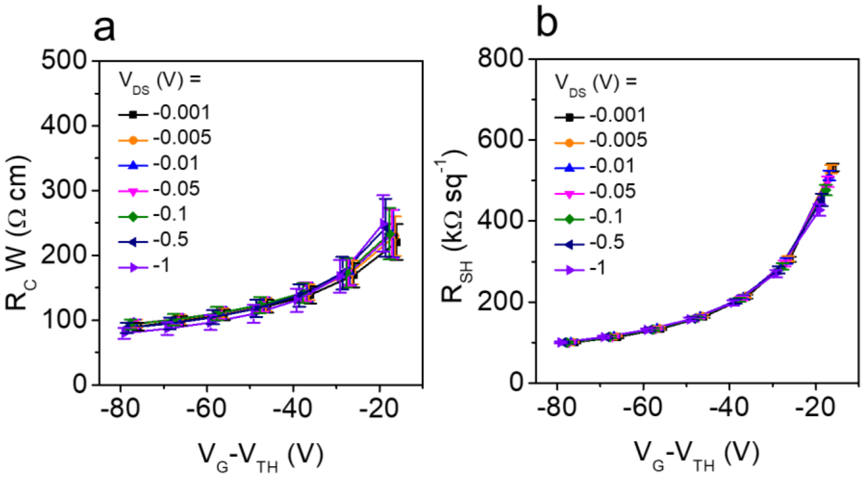

Figure S15. The extracted $R_C W$ and R_{SH} at different V_{DS}. (a) The extracted $R_C W$ at different V_{DS}. (b) The extracted R_{SH} at different V_{DS}.

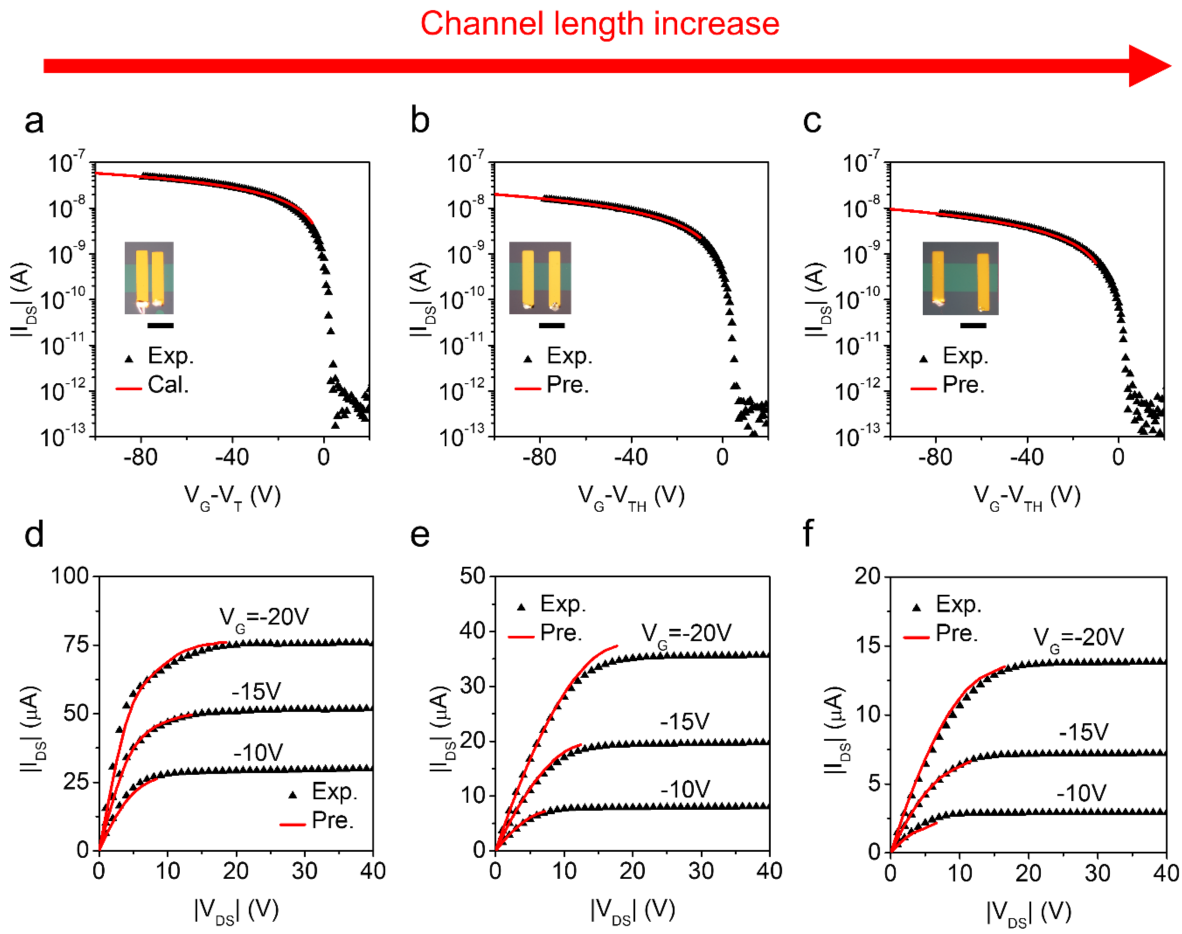

Figure S16. The prediction of electrical performance of monolayer OFET with different channel lengths. The prediction of transfer curves (a to c) and output curves (d to f). The respective channel lengths are (a and d) 12 μm, (b and e) 56 μm and (c and f) 125 μm. The POM images of the devices are shown in the insets in (a to c). The scale bars are 100 μm. The transfer curves were tested with a V_{DS} of -1 mV while the outputs are under the V_G of -10 V, -15 V and -20 V.

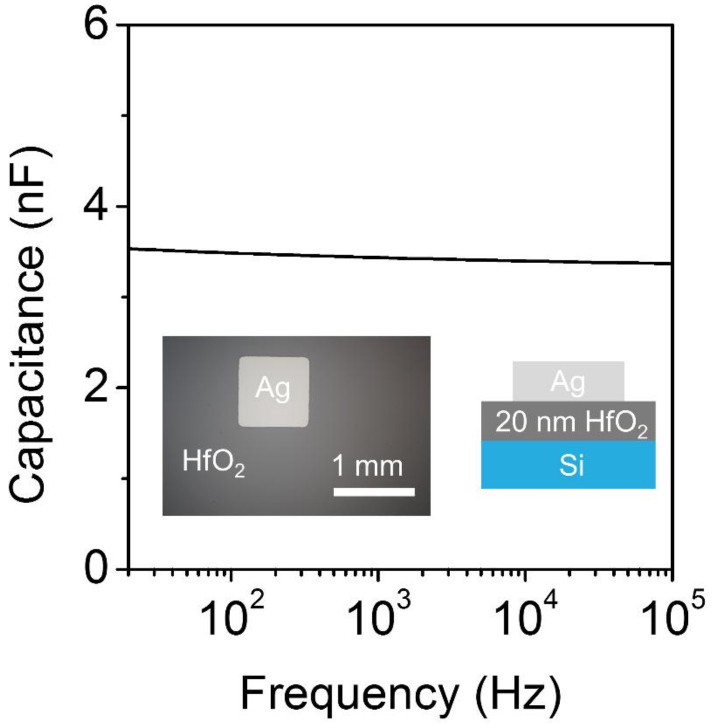

Figure S17. Capacitance measurement on 20 nm HfO_2. The inset shows the optical image and the schematic diagram of the capacitance structure, in which the HfO_2 is sandwiched by the Ag and the Si. The scale bar is shown in the optical image.

References

[1] Y. Liu, J. Guo, E. Zhu, L. Liao, S.-J. Lee, M. Ding, I. Shakir, V. Gambin, Y. Huang, X. Duan, *Nature* **2018**, *557*, 696.
[2] S. Ding, Y. Tian, H. Wang, Z. Zhou, W. Mi, Z. Ni, Y. Zou, H. Dong, H. Gao, D. Zhu, *ACS Nano* **2018**, *12*, 12657.
[3] K. Jung, Y. C. Kim, B. Park, H. Shin, J. D. Lee, *IEEE Trans. Electron Devices* **2009**, *56*, 431.
